# Clinical Anatomy of the Cavotricuspid Isthmus and Terminal Crest

**DOI:** 10.1371/journal.pone.0163383

**Published:** 2016-09-28

**Authors:** Wiesława Klimek-Piotrowska, Mateusz K. Hołda, Mateusz Koziej, Jakub Hołda, Katarzyna Piątek, Kamil Tyrak, Filip Bolechała

**Affiliations:** 1 Department of Anatomy, Jagiellonian University Medical College, Cracow, Poland; 2 Department of Forensic Medicine, Jagiellonian University Medical College, Cracow, Poland; Boston University, UNITED STATES

## Abstract

The aim of this study was to provide useful information about the cavotricuspid isthmus (CTI) and surrounding areas morphology, which may help to plan CTI radio-frequency ablation. We examined 140 autopsied human hearts from Caucasian individuals of both sexes (29.3% females) with a mean age of 49.1±17.2 years. We macroscopically investigated the lower part of the right atrium, the CTI, the inferior vena cava ostium and the terminal crest. The paraseptal isthmus (18.5±4.0 mm) was significantly shorter than the central isthmus (p<0.0001), and the central isthmus (24.0±4.2 mm) was significantly shorter than the inferolateral isthmus (29.3±4.9 mm) (p<0.0001). Heart weight was positively correlated with all isthmus diameters. Three different sectors of CTI were distinguished: anterior, middle and posterior. The middle sector of the CTI presented a different morphology: trabeculae (N = 87; 62.1%), intertrabecular recesses (N = 35; 25.0%) and trabecular bridges (N = 18; 12.9%). A single sub-Eustachian recess was present in 48.6% of hearts (N = 68), and a double recess was present in 2.9% of hearts (N = 4) with mean depth = 5.6±1.8mm and diameter = 7.1±3.4mm. The morphology of the distal terminal crest was varied; 10 patterns of the distal terminal crest ramifications were noted. There were no statistically significant differences in any of the investigated CTI parameters between groups with different types of terminal crest ramifications. The presence of intertrabecular recesses (25.0%), trabecular bridges (12.9%) and sub-Eustachian recesses (48.6%) within the CTI can make ablation more difficult. We have presented the macroscopic patterns of final ramifications of the terminal crest within the quadrilateral CTI area.

## Introduction

The cavotricuspid isthmus (CTI) is a part of the right atrium located between the inferior vena cava (IVC) ostium and the tricuspid valve. The CTI is a relatively new concept that was first introduced by Cosio et al. (1993); this region of the heart plays an essential role in the atrial flutter circuit [1]. Since then, this small, quadrilateral-shaped area of the right atrium has served as a target for catheter-directed ablation, which has become the method of choice for treating atrial flutter [2]. Despite very high success rates and almost no complications [3], ablation of the CTI can be extremely difficult in some patients with atypical anatomical conditions; the CTI anatomy is complex and associated with a significant inter-individual variability [4–6].

Knowledge of the detailed anatomy of this region can significantly improve the safety and success rate of ablation procedures. The morphology and muscular architecture of the CTI in the human heart, regardless of its relevance to clinical practice, is not yet fully understood. There have been only a few anatomical studies on this topic, and only three of them comprehensively investigated the majority of CTI dimensions [4, 5, 7–11]. Therefore, the aim of this study is to provide more information about the morphology of the CTI and surrounding areas in structurally normal hearts. We studied the macroscopic muscular architecture of the lower-right atrium area to provide anatomical details relevant to clinical practice.

## Materials and Methods

### Study population

This study was conducted in the Department of Anatomy, Jagiellonian University Medical College (Cracow, Poland) and was approved by the Bioethical Committee of Jagiellonian University Medical College (KBET/51/B/2013). In our study we personally collected hearts only from deceased person who did not express objection, when alive, and if family did not express objection. In accordance with Polish Law our Bioethical Committee waived the need for written or verbal informed consent. These samples were not procured from a tissue bank or donation center.

We studied 140 autopsied human hearts from Caucasian individuals of both sexes (29.3% females) with a mean age of 49.1±17.2 years and an average body mass index (BMI) of 27.7±6.1 kg/m^2^ and a mean body surface area of 1.9±0.2 m². We collected the hearts during routine forensic medical autopsies performed in the Department of Forensic Medicine, Jagiellonian University Medical College from July 2013 until November 2015. The primary causes of death were: suicide, murders, traffic accidents and home accidents. The exclusion criteria included severe anatomical defects, heart surgeries or heart grafts, evident severe macroscopic pathologies of the heart or vascular system found during autopsy (aneurysms, storage diseases), heart trauma and macroscopic signs of cadaver decomposition. None of the 140 individuals had a history of any type of arrhythmia.

### Dissection and measurements

The hearts were dissected together with the proximal portions of the great vessels: the ascending aorta, pulmonary trunk, superior vena cava, IVC, and all of the pulmonary veins. We weighed the hearts before fixation using an electronic laboratory scale with a precision of 0.5 g (BSA-L Laboratory). After dissection, all of the hearts were fixed by immersion in 10% paraformaldehyde solution for a maximum of two months until the time of measurement [12].

The right atrium was opened in a routine way using an intercaval incision extending from the orifice of the superior vena cava to the orifice of the IVC without sectioning the orifices. If necessary, additional cuts were made to present the investigated area in a better way. We obtained linear measurements using YATO electronic calipers (YT–7201) precise to 0.03 mm. All of the measurements were made by two independent researchers in order to reduce bias. If the measurement differences between the researchers exceeded 10%, both measurements were repeated. The mean of the two measurements was calculated and approximated to a tenth of a decimal place.

**Fig 1:**
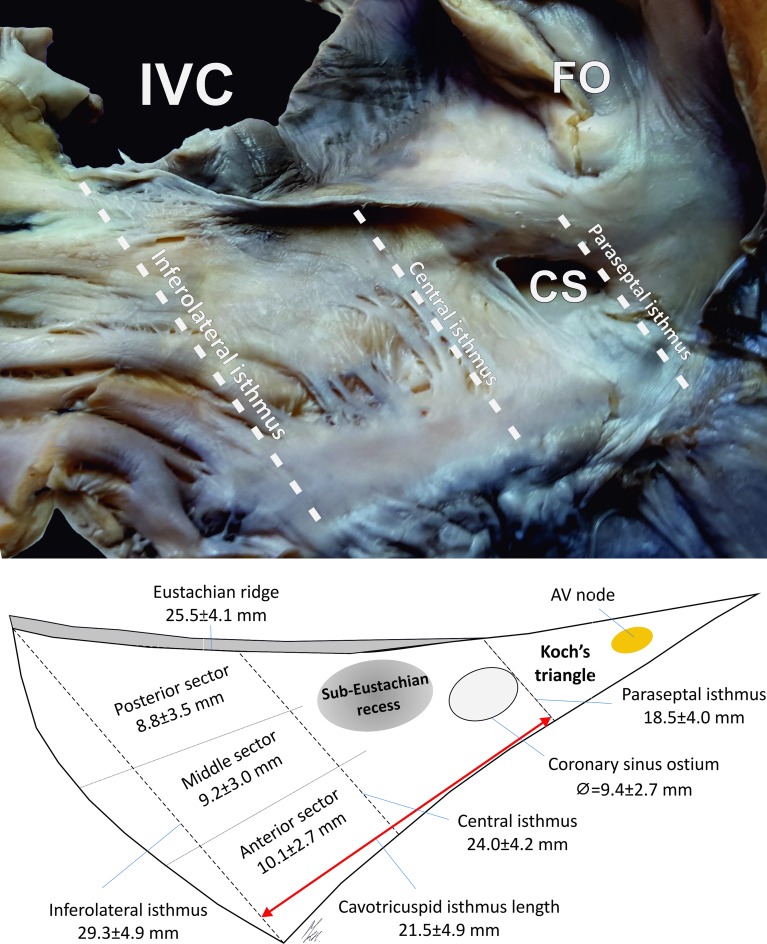
Photograph of a cadaveric heart specimen showing the cavotricuspid isthmus area and a schematic view of the investigated heart region (mean ± standard deviations). AV–atrioventricular; CS–coronary sinus ostium; FO–fossa ovalis; IVC–inferior vena cava.

The lower part of the right atrium, CTI, IVC ostium and terminal crest (TC) were investigated. The CTI is the quadrilateral-shaped area bounded medially by the paraseptal isthmus, laterally by the inferolateral isthmus, anteriorly by the septal tricuspid leaflet attachment and posteriorly by the Eustachian valve and ridge. The following measurements were made (Fig 1):

- paraseptal isthmus (or septal isthmus/Koch’s triangle base)–width of the closed-line segment tangential to the left contour of the coronary sinus bounded by the point where it touches the tricuspid annulus anteriorly and by the left end of the Eustachian ridge posteriorly;
- central isthmus (or inferior isthmus)–width of the closed-line segment bounded by the point where it touches the septal leaflet anteriorly and by the midpoint of the Eustachian ridge posteriorly, parallel to the paraseptal isthmus;
- inferolateral isthmus–width of the closed line segment bounded by the right end of the septal leaflet anteriorly and by the right end of the Eustachian ridge posteriorly, parallel to the paraseptal and central isthmus;
- the diameter of the IVC ostium;
- the height of the Eustachian valve, measured as the length between the free edge of the valve and its attachment site to the right atrium;
- the length of the Eustachian ridge between the paraseptal and inferolateral isthmus;
- the total length of the septal tricuspid leaflet attachment;
- the shortest length between the paraseptal and inferolateral isthmus (CTI length);
- the thickness of the Eustachian ridge;
- the depth and diameters of the sub-Eustachian recesses;
- the diameter of the coronary sinus ostium;
- the diameter of the right atrioventricular ring.

We distinguished three morphological sectors within the CTI (between the central and inferolateral isthmus): anterior, middle and posterior. The antero-posterior dimensions of the parts noted above at the level of the central isthmus were measured, and their morphology were evaluated. We also calculated the CTI area. The presence of the sub-Eustachian recess (also known as the sub-Thebesian recess or the sinus of Keith) was assessed.

We used transillumination to macroscopically evaluate patterns of the final ramifications of the distal TC muscle fibers into the lower part of the right atrium.

### Statistical analysis

The data are presented as mean values with the corresponding standard deviations or percentages. We performed statistical analyses with STATISTICA v12 (StatSoft Inc., Tulsa, OK, USA). A p value of less than 0.05 was considered to be statistically significant. The Shapiro-Wilk test was used to determine if the quantitative data were normally distributed. To verify homogeneity of variance, we performed Levene’s test. We also used the Student’s *t*-tests and the Mann-Whitney U tests for statistical comparisons. We performed Kruskal-Wallis one-way analysis of variance to determine significant differences in the investigated CTI parameters between groups with different types of TC ramifications. Correlation coefficients were calculated to measure the statistical dependence between the measured hearts’ parameters with scatter plots generated for selected cases.

## Results

**Fig 2:**
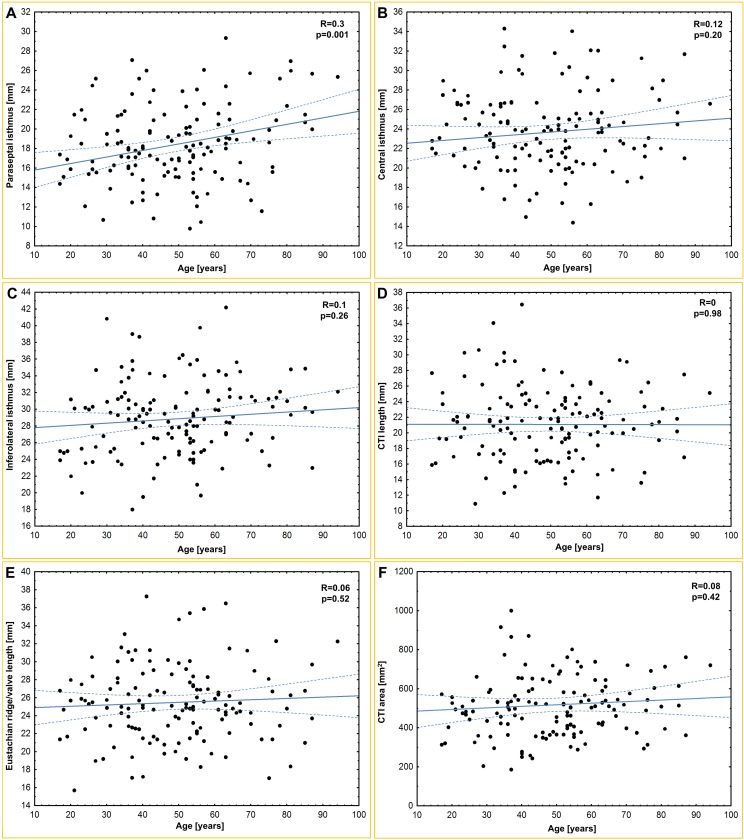
Scatter plots of age and (A) paraseptal isthmus; (B) central isthmus; (C) inferolateral isthmus; (D) cavotricuspid isthmus (CTI) length; (E) Eustachian ridge/valve length; (F) CTI area.

**Fig 3:**
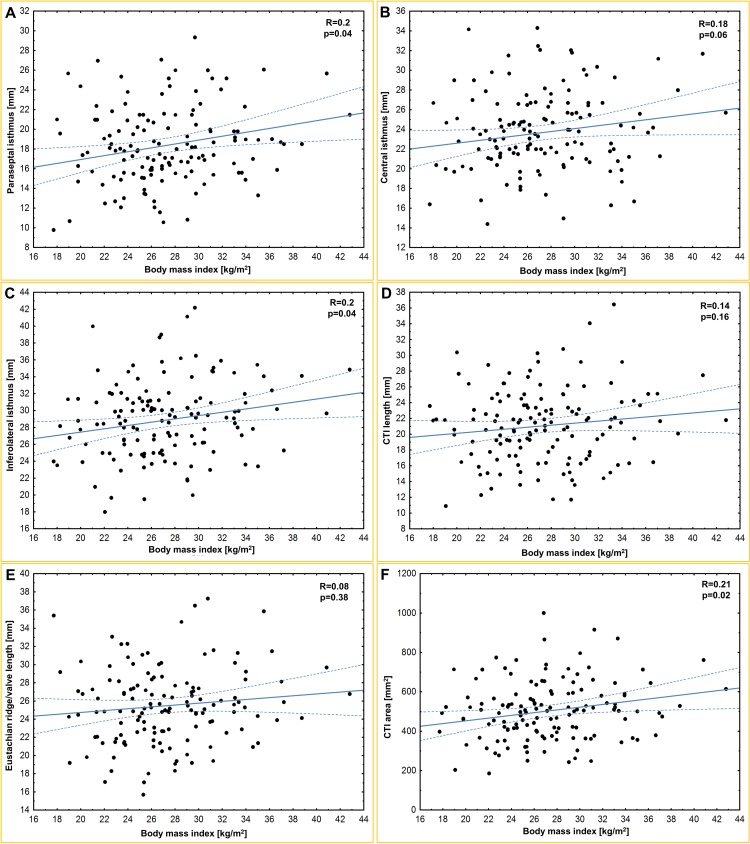
Scatter plots of body mass index (BMI) and (A) paraseptal isthmus; (B) central isthmus; (C) inferolateral isthmus; (D) cavotricuspid isthmus (CTI) length; (E) Eustachian ridge/valve length; (F) CTI area.

**Fig 4:**
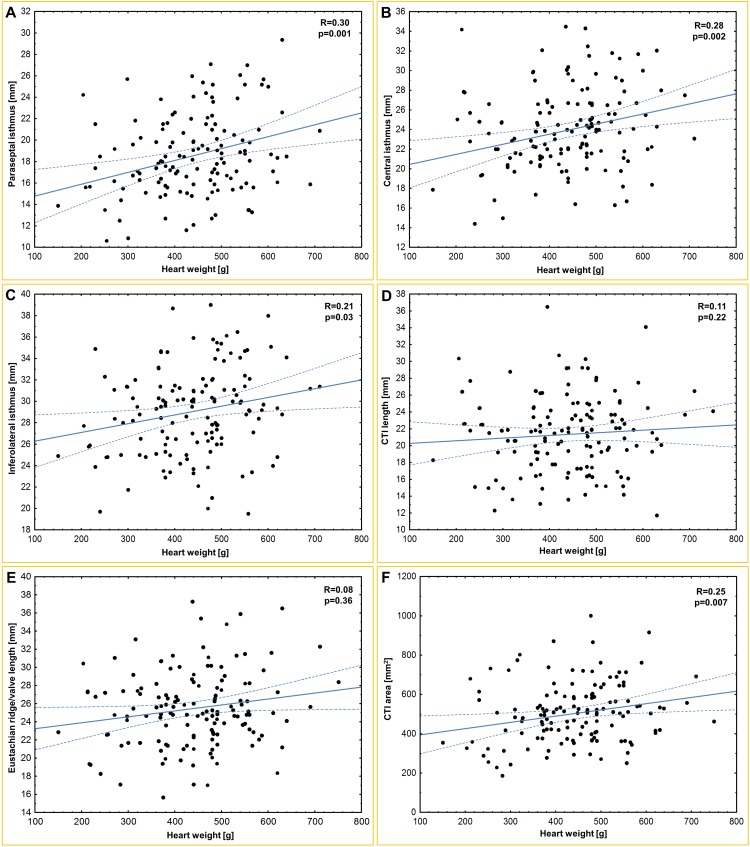
Scatter plots of heart weight and (A) paraseptal isthmus; (B) central isthmus; (C) inferolateral isthmus; (D) cavotricuspid isthmus (CTI) length; (E) Eustachian ridge/valve length; (F) CTI area.

The mean heart weight was 441.0 ± 119.1 g. Table 1 and Fig 1 present the results of all of the obtained measurements. The paraseptal isthmus was significantly shorter than the central isthmus (p<0.0001), and the central isthmus was significantly shorter than the inferolateral isthmus (p<0.0001). The heart weight was correlated with all of the isthmus dimensions: paraseptal (r = 0.30; p = 0.001), central (r = 0.28; p = 0.002) and inferolateral (r = 0.21; p = 0.03). Furthermore, we found that a larger coronary sinus ostium diameter was correlated with a longer paraseptal (r = 0.25; p = 0.01), central (r = 0.20; p = 0.04) and inferolateral (r = 0.23; p = 0.01) isthmus. The paraseptal isthmus width was positively correlated with age (r = 0.3; p<0.001) and BMI (r = 0.2; p = 0.04). The inferolateral isthmus was positively correlated with the BMI (r = 0.2; p = 0.04). The CTI length was positively correlated with the diameter of the right atrioventricular ring (r = 0.25; p = 0.01). The CTI surface area was positively correlated with BMI (r = 0.21; p = 0.02), heart weight (r = 0.25; p = 0.007) and Eustachian valve height (r = 0.26; p = 0.02). Scatter plots of age, BMI and heart weight for selected CTI dimensions are presented in Figs 2–4. There were no significant differences in any of the measured CTI diameters between the sexes (Table 2).

**Table 1:** Results of obtained measurements.

	N	Mean	SD	Min	Max	Median	Q_1_	Q_3_
**Age [years]**	140	49.1	17.2	17.0	94.0	50.0	37.0	60.0
**BMI [kg/m**^**2**^**]**	140	27.7	6.1	17.7	50.6	26.2	23.7	29.9
**Heart weight [g]**	140	441.0	119.1	150.0	750.0	440.0	372.3	524.8
**Paraseptal isthmus [mm]**	140	18.5	4.0	9.8	29.4	18.0	15.9	21.0
**Central isthmus [mm]**	140	24.0	4.2	14.4	35.1	23.8	21.3	26.5
**Inferolateral isthmus [mm]**	140	29.3	4.9	18.0	51.8	29.3	26.2	31.4
**CTI length [mm]**	140	21.5	4.9	10.9	39.0	21.5	18.7	23.7
**Inferior vena cava ostium [mm]**	140	25.0	5.6	9.1	40.1	24.7	21.4	28.8
**Eustachian valve height [mm]**	99	5.0	2.3	1.0	11.4	4.9	3.1	6.6
**Eustachian ridge/valve length [mm]**	140	25.5	4.1	15.7	37.3	25.1	22.7	27.5
**Eustachian ridge thickness [mm]**	67	3.6	1.9	1.2	8.5	2.9	2.1	4.5
**Septal leaflet attachment length [mm]**	140	39.2	7.2	24.8	61.2	34.9	39.1	42.3
**Sub-Eustachian recces diameter [mm]**	72	7.1	3.4	3.1	17.9	8.1	5.9	10.6
**Sub-Eustachian recces depth [mm]**	72	5.6	1.8	2.1	10.8	5.5	4.6	6.9
**Right atrioventricular ring diameter [mm]**	140	28.9	4.7	17.1	42.7	28.5	25.8	31.6
**Coronary sinus ostium diameter [mm]**	140	9.4	2.7	4.0	18.3	9.0	7.5	11.0
**CTI anterior sector dimension [mm]**	140	10.1	2.7	3.3	21.5	10.1	8.6	11.6
**CTI middle sector dimension [mm]**	140	9.2	3.0	3.3	19.1	8.9	7.2	11.3
**CTI posterior sector dimension [mm]**	140	8.8	3.5	1.9	17.3	8.2	6.2	10.4
**CTI area [mm**^**2**^**]**	140	517.3	181.5	187.6	1295.2	504.3	398.8	594.8

BMI–body mass index, CTI–cavotricuspid isthmus, N–number of samples, SD–standard deviation, Q_1_ and Q_3_—lower and upper quartiles

**Table 2:** Data distribution by sex–no statistically significant differences were observed except for the heart weight.

	Total	Males			Females			p
	N	N	Mean	SD	N	Mean	SD	
**Age [years]**	140	99	48.2	15.7	41	51.6	20.9	0.347
**BMI [kg/m**^**2**^**]**	140	99	27.1	4.1	41	28.2	7.4	0.891*
**Heart weight [g]**	140	99	475.9	99.3	41	397.6	108.3	0.000*
**Paraseptal isthmus [mm]**	140	99	18.6	4.1	41	18.3	3.7	0.695
**Central isthmus [mm]**	140	99	24.1	4.3	41	23.0	3.5	0.179
**Inferolateral isthmus [mm]**	140	99	29.3	4.9	41	28.7	4.6	0.760*
**CTI length [mm]**	140	99	21.3	4.2	41	21.6	6.5	0.702*
**Inferior vena cava ostium [mm]**	140	99	25.1	5.7	41	24.4	5.6	0.730
**Eustachian valve height [mm]**	99	69	5.2	2.3	30	5.0	2.4	0.650
**Eustachian ridge/valve length [mm]**	140	99	25.5	4.3	41	25.8	3.8	0.346*
**Eustachian ridge thickness [mm]**	67	42	3.7	2.1	23	3.4	1.4	0.934*
**Septal leaflet attachment length [mm]**	140	99	39.4	6.8	41	38.2	6.2	0.685
**Sub-Eustachian recces diameter [mm]**	72	51	8.9	3.7	21	7.9	2.4	0.334
**Sub-Eustachian recces depth [mm]**	72	51	5.7	1.9	21	5.3	1.6	0.496
**Right atrioventricular ring diameter [mm]**	140	99	28.7	4.7	41	29.5	4.5	0.458
**Coronary sinus ostium diameter [mm]**	140	99	9.5	2.8	41	9.0	2.5	0.414
**CTI anterior sector dimension [mm]**	140	99	10.2	2.7	41	9.8	2.6	0.591*
**CTI middle sector dimension [mm]**	140	99	9.2	3.2	41	9.5	4.9	0.453*
**CTI posterior sector dimension [mm]**	140	99	9.1	4.2	41	9.3	3.4	0.604*
**CTI area [mm**^**2**^**]**	140	99	515.3	165.9	41	522.4	220.5	0.910*

*—non-parametric

BMI–body mass index, CTI–cavotricuspid isthmus, N–number of samples, SD–standard deviation, Q_1_ and Q_3_—lower and upper quartiles

**Fig 5:**
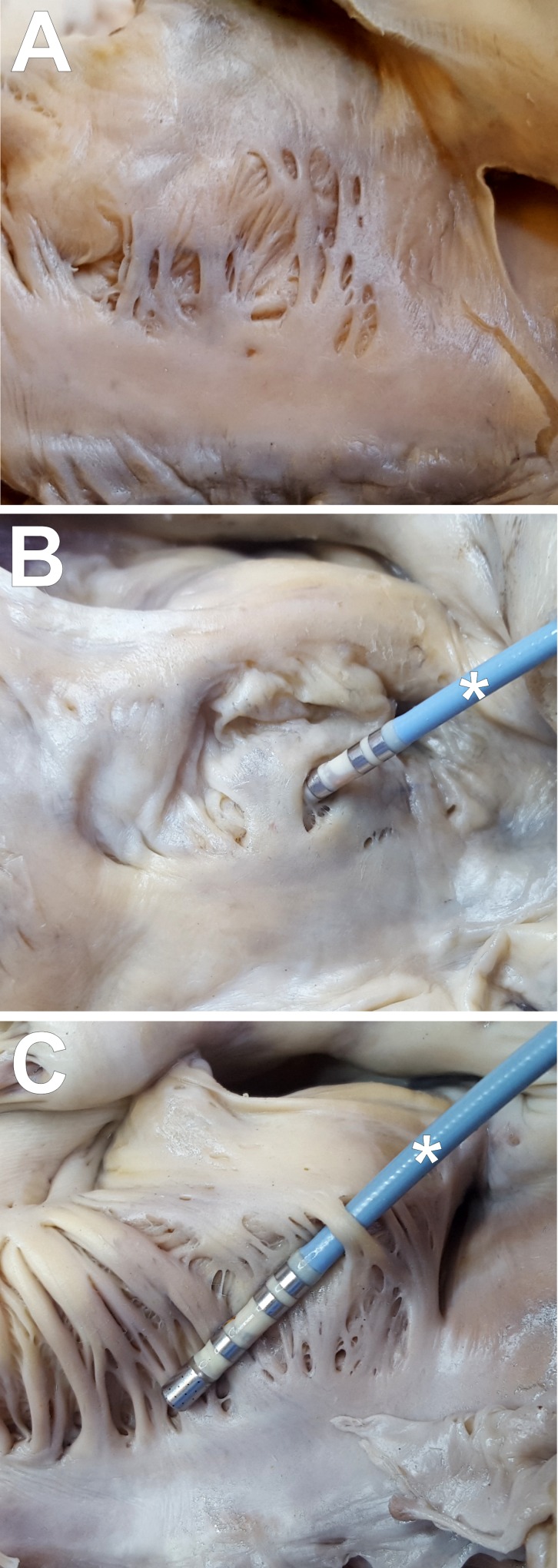
Three different types of muscular arrangement in the middle sector of the cavotricuspid isthmus. The anatomical position of samples has not been maintained during the taking photos to get a better picture of cavotricuspid isthmus sectors. (A) trabeculae (N = 87; 62.1%); (B) intertrabecular recesses (N = 35; 25.0%); (C) trabecular bridges (N = 18; 12.9%); *–electrocardiological catheter.

We distinguished three different sectors of CTI between the central and inferolateral isthmus: anterior (smooth), middle (trabeculated) and posterior (membranous) (Table 1). The middle sector of the CTI presented a different morphology: trabeculae (N = 87; 62.1%), intertrabecular recesses (N = 35; 25.0%) and trabecular bridges (N = 18; 12.9%) (Fig 5). The mean ratio of the middle sector width to the central isthmus width was 0.3 ± 0.1. We found that the width of the anterior sector increased with age (r = 0.26; p = 0.009).

The Eustachian valve was present in 70.7% of hearts (N = 99). Its height was positively correlated with the width of the inferolateral isthmus (r = 0.21; p = 0.05). When the Eustachian valve was present, the central and inferolateral isthmus were significantly longer compared with specimens without the valve (p = 0.0.3 and p = 0.006, respectively). Moreover, the inferolateral and paraseptal isthmus width were positively correlated with the valve’s height (r = 0.27; p = 0.02 and r = 0.4; p = 0.001, respectively).

**Fig 6:**
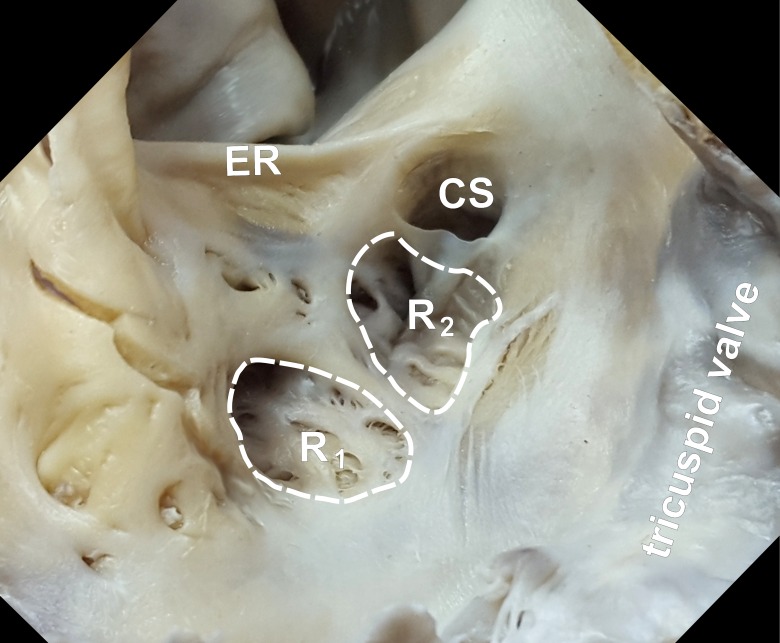
Double sub-Eustachian recess (R_1_ and R_2_). CS–coronary sinus ostium; ER–Eustachian ridge.

The single sub-Eustachian recess was present in 48.6% of hearts (N = 68), and the double recess was present in 2.9% of hearts (N = 4) (Fig 6). All of the recesses were localized between the paraseptal and central isthmus to the right of the coronary sinus ostium or the central isthmus traversing the recess. The prominent Eustachian ridge occurred in 47.9% of hearts (N = 67), and its thickness was negatively correlated with the IVC ostium diameter (r = -0.27; p = 0.04). The diameter of the sub-Eustachian recess was significantly larger in hearts without the Eustachian ridge (p = 0.01) and negatively correlated with the diameter of the right atrioventricular ring (r = -0.31; p = 0.01). The depth of the sub-Eustachian recess was positively correlated with the length of the Eustachian ridge between the paraseptal and inferolateral isthmus (r = 0.24; p = 0.04).

**Fig 7:**
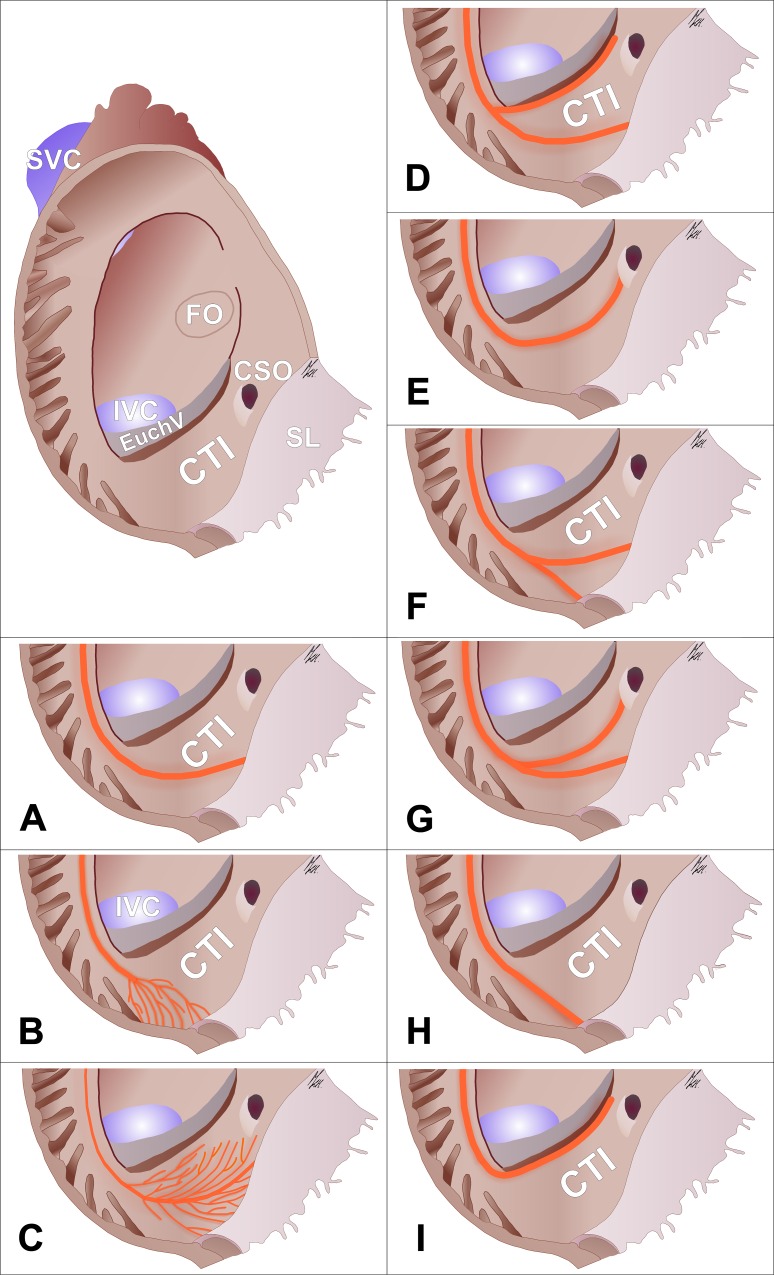
Schemes of the final ramifications of the distal terminal crest muscle fibers (orange) into the lower part of the right atrium (types A–I; see Table 3). Smaller bands branching from the major crest were not presented with the exception of types B and C in which the main muscle band is absent. CSO–coronary sinus ostium; CTI–cavotricuspid isthmus; EuchV–Eustachian valve; FO–fossa ovalis; IVC–inferior vena cava; SL–septal leaflet; SVC–superior vena cava.

We found that the morphology of the distal TC presented considerable variability. Based on transillumination of the lower-right atrium walls, we were able to macroscopically classify the distal TC ramifications into the lower part of the right atrium. The division was made on the basis of the main muscle band course (Table 3). Smaller muscular bands branching from the major crest presented a non-uniform architecture and radiated to the Eustachian ridge, coronary sinus ostium and vestibule of the tricuspid valve. Fig 7 presents schemes of all of the established TC types. There were no statistically significant differences in any of the investigated CTI parameters between groups with different types of TC ramifications.

**Table 3:** Patterns of the final ramifications of the terminal crest muscle fibers into the lower part of the right atrium.

Type	N	%	Description
**A**	36	25.7%	Thick muscular bundle from the distal crest to the vestibule of the tricuspid valve
**B**	22	15.7%	Many thinner bundles radiated from the distal crest beyond the CTI (to the right)
**C**	20	14.3%	Bundles of the distal crest radiated in fan-like fashion, obliquely in the CTI area
**D**	10	7.1%	Two thick muscular bundles from distal crest to the vestibule of the tricuspid valve and to the Eustachian ridge
**E**	9	6.4%	Thick bundle extended obliquely through CTI to the coronary sinus ostium, below the Eustachian ridge/valve
**F**	8	5.7%	Two thick muscular bundles from distal crest to the vestibule of the tricuspid valve going through and beyond the CTI
**G**	7	5.0%	Two thick muscular bundles from distal crest to the vestibule of the tricuspid valve and to the coronary sinus ostium
**H**	5	3.6%	Thick muscular bundle extending to the tricuspid valve beyond the CTI
**I**	3	2.1%	Thick muscular bundle from the distal crest to the Eustachian ridge
**J**	20	14.3%	The combination of the above mentioned patterns

CTI–cavotricuspid isthmus, N- number of samples

## Discussion

There have only been three anatomical studies that have comprehensively investigated the majority of CTI dimensions [4, 6, 7]. Pejkovic and Krajnc as well as Gami et al. have investigated precisely the CTI region, but these authors did not measure the major CTI dimensions [9, 13]. Table 4 presents a comparison our results with those of previous studies. A fundamental difference concerns the width of the central isthmus. Cabrera et al. showed that the central isthmus is the shortest of all isthmus dimensions; it likely represents the best of all possible ablation sites [4]. We cannot confirm this finding. We found that the paraseptal isthmus was significantly shorter than the central isthmus. Our results, however, are consistent with imaging study observations that were obtained using multi-detector row computed tomography; these observations revealed that the central isthmus was also the shortest (Table 4) [6].

**Table 4:** Comparison between results of the present study and those conducted previously (mean ± standard deviation).

	Present study	Cabrera et al. (1998) [7]	Cabrera et al. (2005) [4]	Saremi et al. (2008) [6]
**N**	140	28	30	201
**Type of materials**	cadavers	cadavers	cadavers	MDR-CT*
**Paraseptal isthmus [mm]**	18.5±4.0	26.4±4.0	24.0±4.0	20.0±3.5
**Central isthmus [mm]**	24.0±4.2	-	19.0±4.0	24.0±4.3
**Inferolateral isthmus [mm]**	29.3±4.9	31.0±4.0	30.0±3.0	27.0±4.8
**Eustachian ridge/valve length [mm]**	25.5±4.1	31.0±5.0	-	-
**Prominent Eustachian ridge**	N = 67 (47.9%)	N = 7 (25%)	N = 8 (26.7%)	N = 49 (24%)
**Eustachian ridge thickness [mm]**	3.6±1.9	-	3.2±0.8	2.8±1.8
**Sub-Eustachian recces**	single—N = 68 (48.6%); double—N = 4 (2.9%)	-	N = 25 (80%)	N = 114 (56.7%)
**Sub-Eustachian recces diameter [mm]**	7.1±3.4	-	14.0±3.0	7.3 ±2.3
**Sub-Eustachian recces depth [mm]**	5.6±1.8	-	2.9±1.2	7.7±2.6
**Coronary sinus ostium diameter [mm]**	9.4±2.7	9.5±2.0	-	9.1±2.0
**CTI anterior sector dimension [mm]**	10.1±2.7	12.0±2.0	-	-
**CTI middle sector dimension [mm]**	9.2±3.0	8.0±4.0	-	-
**CTI posterior sector dimension [mm]**	8.8±3.5	10.0±4.0	-	-

*middiastole (70% of Cardiac Cycle)

CTI–cavotricuspid isthmus; MDR-CT–multi-detector row computed tomography, N–number of samples

Although not the shortest, the inferolateral and central isthmuses are considered to be better places for ablation [14]. Ablation at the paraseptal isthmus is associated with a significant risk of atrioventricular node injury and conduction block. Moreover, ablation difficulties are not typically caused by anatomical width; they are instead caused by tissue thickness, pouches or muscular bridges or trabeculae [15]. The paraseptal region has the thickest muscular content among the CTI [4]. Therefore, despite its shortest width, it is not a preferable target for ablation.

The TC plays an important role in typical atrial flutter. It provides a barrier to conduction transversely across it. The transverse conduction block of the TC occurs more likely in thick bundles. On the other hand, fast conduction velocities can be observed in the longitudinal direction of the TC (anisotropy) [16]. The proximal and intercaval course of the TC is quite universal in all hearts [8]. The distal TC (final ramifications) and its non-uniform pattern seems to play a role in the propagation of impulses and has an impact on the success rate of CTI ablation [17]. In this study, we present the primary patterns of TC final ramifications within the CTI (Table 3, Fig 7). Conduction in the CTI courses preferentially along thicker bundles that could become the targets for ablation. The main pattern represents one thick bundle of the distal crest terminating in the vestibule of the tricuspid valve (26%), followed by many thinner bundles radiating from the distal crest either beyond the CTI (16%) or obliquely in the CTI area in a fan-like fashion (14%). From our point of view, the most interesting types are those in which the TC continues its course as a thick muscular bundle through the CTI area (types D, E, G, I). The TC final bundles, according to the so-called “muscle bundle”hypothesis for CTI conduction, can serve as preferential conduction pathways [15]. Ablation of significantly thicker TC in lineal CTI ablation may necessitate increased radio-frequency energy and be the cause of procedural failure. On the other hand, the punctual ablation of distal CT may result in the interruption of pathological conduction. Clinical studies devoted to the role of different types of TC ramifications are clearly required.

The size of the Eustachian valve and ridge may also affect negatively CTI ablation [18]. These parameters may hinder catheter access to the regions located anterior to the ridge and valve. Also, the Eustachian ridge, due to the presence of muscle fibers [5], is able to conduct electrical impulses and represents the site of conduction gaps that are difficult to ablate. In these cases, only the complete abolition of the Eustachian ridge using more powerful ablation catheters results in complete bidirectional isthmus block [19]. We have demonstrated the continuation of the TC in the Eustachian ridge in nearly 10% of all cases (type D and I), which can promote the propagation of impulses.

Sub-Eustachian recesses are common findings with a prevalence reaching 80% [4]. The presence of sub-Eustachian recesses significantly prolongs ablation time and is associated with a higher risk of complications and a lower rate of success [20]. These recesses are always found slightly to the right of the coronary sinus ostium and are never found in the lateral third of the CTI. As a result, the ablation line may be performed more laterally to avoid recesses [13]. On the other hand, we can find intertrabecular recesses (25%) and trabecular bridges (13%) laterally to the sub-Eustachian recesses located in the middle CTI sector. Their transverse arrangement in relation to the long CTI axis (perpendicular to the septal leaflet attachment point) may contribute to enter the catheter between the tissue bridge and the thin atrial wall. The gaps between the bridges and the proper atrial wall may negatively influence the ablation, and more energy may be required to reach the isthmus block.

The majority of anatomical obstacles are unfortunately detected only at the time of CTI ablation, which significantly prolongs the procedure time and reduces the success rate. Meanwhile, even a vague assessment of CTI area contributes significant safety and benefits to pre-procedural ablation by identifying unfavorable anatomy. The CTI can be imaging using computed tomography, magnetic resonance imaging, right atrium angiography, and transesophageal, transthoracic or intracardiac echocardiography [18, 19]. In particular, the use of non-invasive, preprocedural transthoracic echocardiography is strongly recommended for patients since it proves the usefulness for predicting outcome of isthmus-dependent atrial flutter ablation [18].

The primary limitation of our study is that all of the measurements were made on autopsied, structurally normal heart specimens that had been fixed in formaldehyde. This fixing might have resulted in some slight changes in the size and shape of the hearts. However, the use of 10% paraformaldehyde did not cause significant changes in the dimensions of the atrial tissue; the dimensions of fixed hearts are similar to those that are unfixed [12]. We cannot also say anything about the behavior and dimension changes of the CTI region within the cardiac cycle. Our investigation was a purely anatomical study without direct electrophysiological correlates; nevertheless, it provides information relevant to clinical practice.
